# Decision making in breast implant selection for breast reconstruction: A mixed-method study among plastic surgeons

**DOI:** 10.1016/j.jpra.2023.10.009

**Published:** 2023-10-21

**Authors:** E.R. Eijsink, J.X. Harmeling, M.A.M. Mureau, E.M.L. Corten

**Affiliations:** Department of Plastic & Reconstructive Surgery, Erasmus MC Cancer Institute, University Medical Center Rotterdam, Rotterdam, the Netherlands

**Keywords:** Clinical decision making, Breast implants, Reconstructive surgical procedures, Decision making, Shared, Qualitative research, Health care surveys

## Abstract

**Background:**

Breast implants and the (dis)advantages of their characteristics (shape, filling, surface, and brand) have been studied extensively. When selecting a specific breast implant, a plastic surgeon makes a trade-off between the various (dis)advantages. However, the factors affecting the choice of their preferred breast implant have not been studied in detail.

**Methods:**

This is a mixed-method study. First, five plastic surgeons were interviewed to identify factors that influence their choice of a breast implant in a reconstructive setting. Second, 42 plastic surgeons were asked to state their preferred implant, weigh the collected factors, and indicate when they would deviate from their preferred implant.

**Results:**

The interviews produced a varied list of factors that influenced the choice of breast implant, including complication rates, marketing, economic, and logistic factors. The results from the survey showed variation in preferred implant and substantial variations in the weighing of these factors. The two most important factors were “study outcomes” and “brand reputation”. Ninety percent of the respondents were willing to deviate from their preferred implant, with the patient's preference being the main indication to deviate.

**Conclusions:**

The list of factors that influence the plastic surgeons’ choice of a breast implant in a reconstructive setting is extensive and their weighing showed substantial variation. Implant choice was not based solely on scientific evidence. Brand reputation was valued highly, implying that media and marketing may have considerable influence. Therefore, patients must be informed extensively about all aspects of breast implants during shared decision making to obtain true informed consent.

## Introduction

In the Netherlands, with an estimated population of 17 million residents, approximately 30,000 breast implants are inserted annually. One-third of these implants are used for breast reconstruction.^1^ Multiple manufacturers produce a variety of breast implants that differ in shape, filling, and/or surface texture. Plastic surgeons historically aim to find personalized solutions for individual patients. The goal is to choose the right implant for each patient to achieve the best surgical result and optimize patient satisfaction. Procurement of implants varies from country to country. This can be done by hospitals and clinics with limited influence from individual plastic surgeons or by the latter with the free choice of which implant to buy and use. Dutch plastic surgeons often have a significant influence on the procurement and use of implants. Therefore, it would be interesting to know the factors that influence their choice.

In recent years, the plastic surgeon's choice of breast implant type has received increasing media attention. This was partly caused by issues related to breast implant safety, including the reports on the association between breast implant associated anaplastic large-cell lymphoma (BIA-ALCL) and macrotextured breast implants and on breast implant illness (BII), also known as Shoenfeld's syndrome or autoimmune/inflammatory syndrome induced by adjuvants (ASIA).^2^, ^3^, ^4^ This led to the withdrawal of Allergan breast implants from the global market. Furthermore, the involvement of patients in medical decision making has changed over the last few decades. In the past, the patient-physician relationship was more paternalistic, with the doctor deciding what was best for the patient. Nowadays, it has become a standard practice to involve the patient substantially and promote shared decision making and informed consent.^5^^,^^6^

Breast implants and their characteristics have been studied extensively, and certain advantages and disadvantages are attributed to the various implant characteristics. When selecting a specific breast implant type for a patient, one must adjust for the various (dis)advantages that are part of their risk profile. Unfortunately, the literature is inconclusive on what type of implant is the best.^7^ Therefore, preferences of the plastic surgeons differ with regard to implant characteristics. Previous studies have focused on implant size selection or provided consensus recommendations on implant selection in breast augmentation, and also focused specifically on matching implant characteristics to specific patient characteristics.^8^^,^^9^ Other than patient-related characteristics, implant choice might also be influenced by economic, logistic, and other factors. To date, no study has been carried out on how plastic surgeons choose a breast implant for breast reconstruction by considering all possible influencing factors.

Therefore, this study aimed to identify all factors that influence a plastic surgeon's decision or choice of a breast implant and how plastic surgeons weigh these factors.

## Methods

This is a mixed-method study consisting of a qualitative interview study where all factors influencing the choice of a preferred breast implant in a reconstructive setting were collected to develop an online survey to quantitatively evaluate these factors in a larger sample size. The qualitative interview study was reported in accordance with the COnsolidated criteria for REporting Qualitative research guideline.^10^

### Qualitative interview study

All factors influencing the choice of a breast implant for breast reconstruction were collected via semi-structured interviews with reconstructive plastic surgeons. Purposive sampling was used to ensure a representative sample of plastic surgeons. The sample consisted of plastic surgeons who differed in age, gender, professional experience, and hospital setting (academic versus non-academic). Prior to the interviews, a topic list and standard set of questions were formulated with inputs from experts (plastic surgeons) and literature (see “Appendix A: Predefined structure for the interviews”), making a semi-rigged structure for the interviews. This structure was checked and approved by all authors. The interview was pilot tested on a resident in plastic surgery and final adjustments were made to the structure. Next, five plastic surgeons were approached for the interview, all of whom consented after they were informed about this study and its purpose. Preferably, the interviews were carried out in person, but when this was not possible, phone interview was carried out. Personal data was treated in accordance with the general data protection regulation. All sessions were audiotaped, transcribed verbatim, and pseudonymized by Eijsink. This researcher had no experience with breast implants, which reduced the likelihood of bias and/or assumptions. A predefined extraction form was created in advance to facilitate consistent data extraction. The endpoint of the study was reached when saturation regarding factors influencing choice for a breast implant type was reached (i.e., no new factors came up during the interview) in the last two interviews, which was the case after five interviews. Each transcription was analyzed by the same researcher using the predefined extraction form in Microsoft Word and Excel.

### Quantitative online survey study

The data collected in the interview was used to construct an online survey to investigate the importance of all previously identified factors in choosing a breast implant (see “Appendix B: digital survey”). The survey was divided into four parts. First, participants entered characteristics such as gender, age, years in practice, hospital of training, and preferred implant. Second, they had to indicate the extent to which each previously identified factor influenced their choice for their preferred implant on a 6-point Likert scale (strongly disagree, disagree, slightly disagree, slightly agree, agree, and strongly agree). Third, participants ranked three factors that were most important in determining their preference for each specific implant characteristic, i.e., shape, filling, surface, and brand. Finally, the participants were presented with the list of indications for which the interviewees in the pilot study would deviate from their preferred implant. For each indication, respondents were asked if they agreed, and if so, which implant characteristics they would change.

Participation in the survey was anonymous. The survey consisted of mandatory questions, closed options, and an absence of neutral options. The survey was set up in REDCap (version 6.17.2 © 2018 Vanderbilt University) and sent to all 320 members of the Netherlands Society of Plastic Surgery (NVPC).^11^ A reminder was sent after 2 weeks, after which another 2 weeks were given to complete the survey.

Data analysis was performed using Microsoft Word and Excel. Figures were created in either Microsoft Word or Excel, or R studio. Continuous variables are presented as mean and standard deviation (SD). Discrete variables are presented as absolute numbers and percentages.

## Results

### Qualitative interview study

Five interviews were conducted in the period from July to August 2018. The interviews lasted 38 min on average (range: 30–54 min). The plastic surgeons (two females and three males) had an average professional experience of 5.4 years and performed an average of 36 implant-based breast reconstructions per year. Four plastic surgeons also performed augmentation. The surgeons were predominantly employed in general hospitals, two plastic surgeons worked in an academic setting. Their preferred implant characteristics are listed in Table 1. All factors influencing the choice of a specific breast implant that were mentioned in the interviews are listed in Table 2. Table 3 contains indications or motives for which the plastic surgeons would deviate from their preferred implant.

**Table 1:** Implant preferred by the interviewed plastic surgeons for breast reconstruction.

Preferred implant		n (%)
*Shape*	Round	1 (20)
	Anatomical	4 (80)
*Filling*	Silicone	5 (100)
	Other	0 (0)
*Surface*	Textured	3 (60)
	Polyurethane coated	2 (40)
	Smooth	0 (0)
*Brand*	Mentor	2 (40)
	Eurosilicone	1 (20)
	Polytech	2 (40)

**Table 2:** Factors influencing the plastic surgeons’ choice for a breast implant for breast reconstruction.

Ease of use: implantation
Ease of use: explantation
Cost of the implant
Study outcomes
Patient's preference
Cosmetic result
Consistency of the implant (form-retaining/feel)
Service of the industry
Reputation of the implant brand
Risk of complications: BIA-ALCL
Risk of complications: rupture
Risk of complications: ASIA/BII
Risk of complications: rotation / displacement
Risk of complications: capsular contracture
Risk of complications: rippling
Decision of the partnership*
Opinions of colleagues in the field
Plastic surgical training
Habit
Limited knowledge of other implants
Bad experience with other implants

⁎ Brand of breast implants was determined by the department, partnership, or hospital BIA-ALCL = Breast Implant Associated Anaplastic Large-Cell Lymphoma; ASIA = Autoimmune/inflammatory syndrome induced by adjuvants; BII = Breast Implant Illness.

**Table 3:** Indications plastic surgeons mentioned as reasons to deviate from their preferred implant.

Rotation / malposition of previous implant
Capsular contracture of previous implant
Preoperative or postoperative radiotherapy
Bilateral breast reconstruction
Using autologous tissue (for example, an LD*-flap/prosthesis reconstruction)
Preference for a fuller cleavage
Presence of another implant in the contralateral breast
Patient's preference

⁎ LD = latissimus dorsi.

### Survey

**Figure 1:**
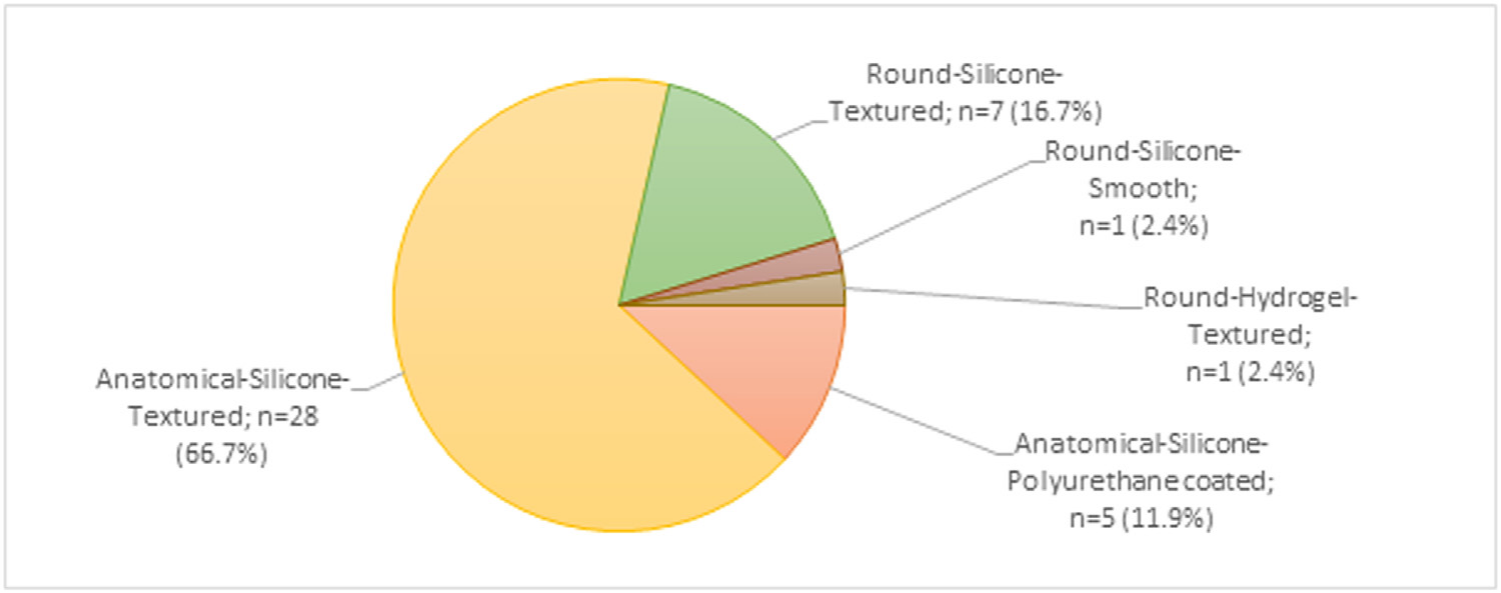
Survey respondents’ preferred breast implant Labels are a combination of the shape, filling, and surface texture of the preferred breast implant.

**Figure 2:**
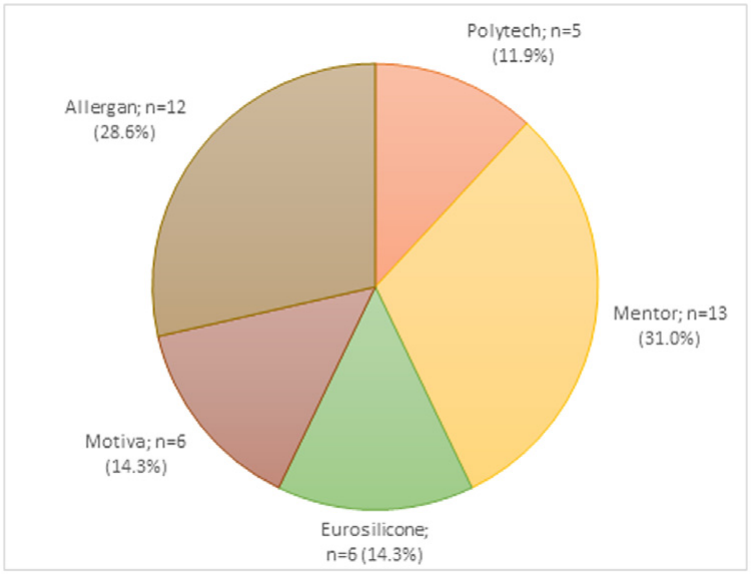
Survey respondents’ preferred brand.

Forty-two plastic surgeons completed the survey from September to October 2018, representing a response rate of 13% of all members of the NVPC.^11^ Plastic surgeons from all Dutch training centers were represented. Respondents had an average age of 47.4 years, average professional experience of 11.7 years, performed an average of 29.6 reconstructions per year, and most of them performed two-stage procedures. Eighty-three percent felt that they could influence the breast implant brand availability in their hospital (Table 4). Figure 1 shows the distribution of our respondents’ preferred implants. Most respondents preferred an implant with a textured surface (86%), silicone filling (98%), and anatomical shape (79%). Among those preferring a textured surface, 56% indicated using a microtexture, 36% used a macrotexture, and 8% did not know what type of texture they used in daily practice. Figure 2 shows the distribution of our respondents’ preferred brands.

**Table 4:** Demographics of survey respondents.

Surgeon characteristics (*n* = 42)		n (%) or mean (SD)
*Sex*	♂	25 (59.5%)
	♀	17 (40.5%)
*Age (years)*		47 (SD=8.3)
*Years in practice*		11.7 (SD=7.3)
*Hospital setting (predominant type)*	Academic	8 (19.0%)
	General	34 (81.0%)
*Breast reconstruction technique (predominately used)*	Direct-to-Implant	10 (23.8%)
	Two-Stage Tissue-Expander	32 (76.2%)
*Also performing augmentation*	No	5 (11.9%)
	Yes, less than reconstruction	20 (47.6%)
	Yes, as much as reconstruction	7 (16.6%)
	Yes, more than reconstruction	10 (23.8%)
*Number of reconstructions performed per year*		29.6 (SD=20.2)
		
*Had influence on the default breast implant selection in their hospital*	Yes	35 (83.3%)
	No	7 (16.7%)

*n* = number; SD = standard deviation.

#### Factors influencing implant preference

**Figure 3:**
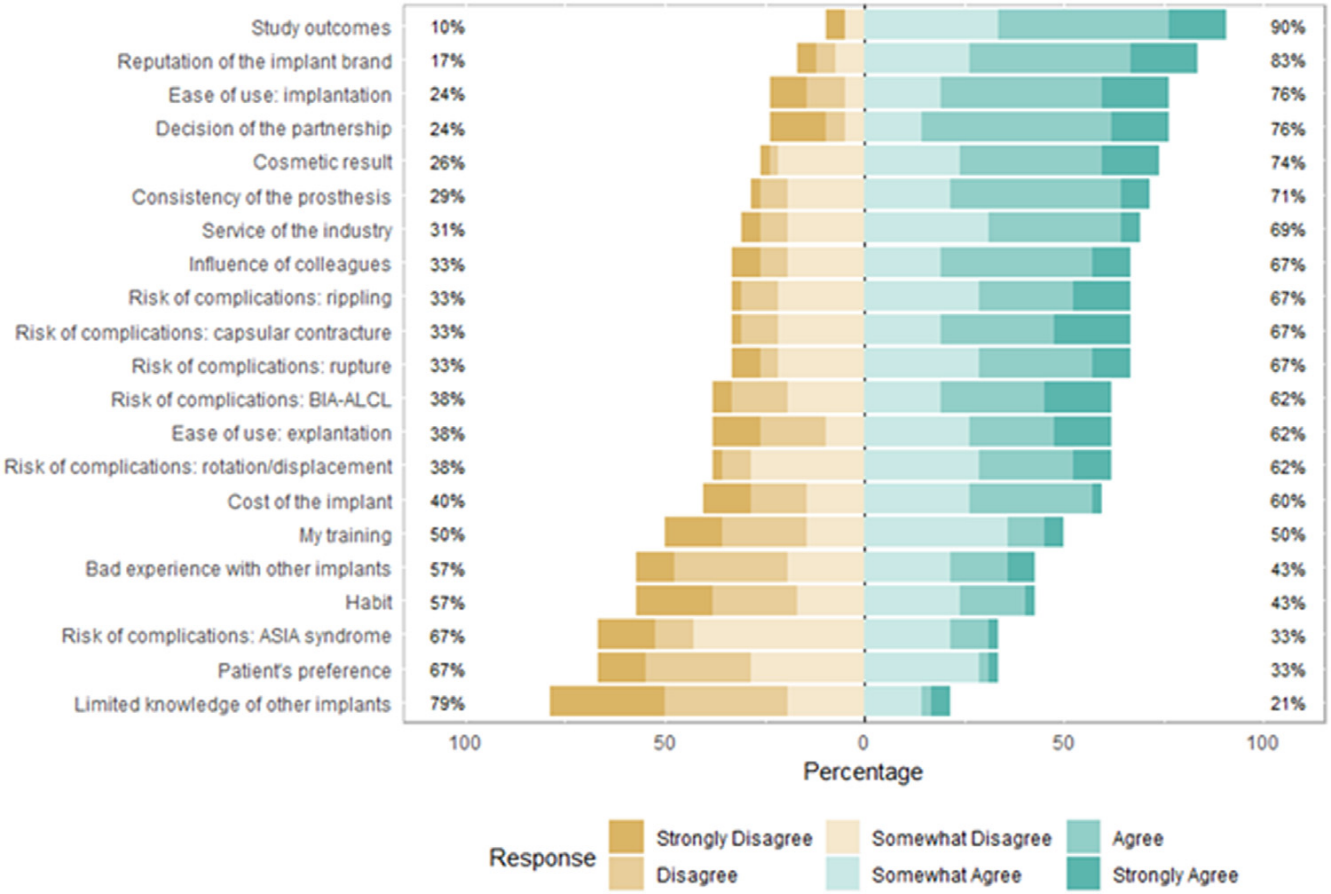
Survey respondents’ weighing the influence of factors in choosing their preferred implant. BIA-ALCL = Breast implant associated anaplastic large-cell lymphoma; ASIA = Autoimmune/inflammatory syndrome induced by adjuvants.

Figure 3 shows how the respondents weighed the influence of each factor in choosing their preferred implant. Sixteen factors were marked as influential by most respondents, with the most critical factors being “study outcomes” (90% overall agreement) and “reputation of the implant brand” (83% overall agreement). Most respondents deemed five factors as unimportant for their choice: “bad experience with other implants,” “habit,” “the risk of ASIA/BII as a complication,” “patient's preference,” and “limited knowledge of other implants.”

#### Ranking factors for separate implant characteristics

**Figure 4:**
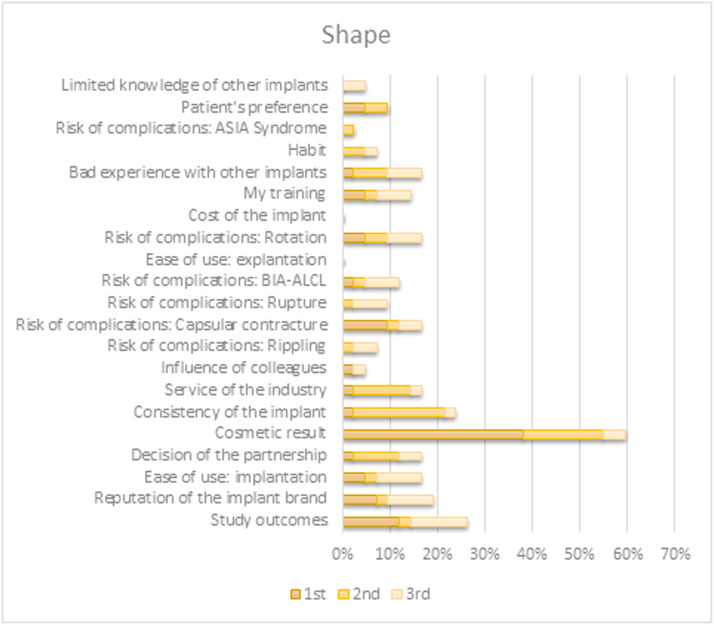
Survey respondents’ first, second, and third most important factor for determining their preferred shape. BIA-ALCL = Breast implant associated anaplastic large-cell lymphoma; ASIA = Autoimmune/inflammatory syndrome induced by adjuvants.

**Figure 5:**
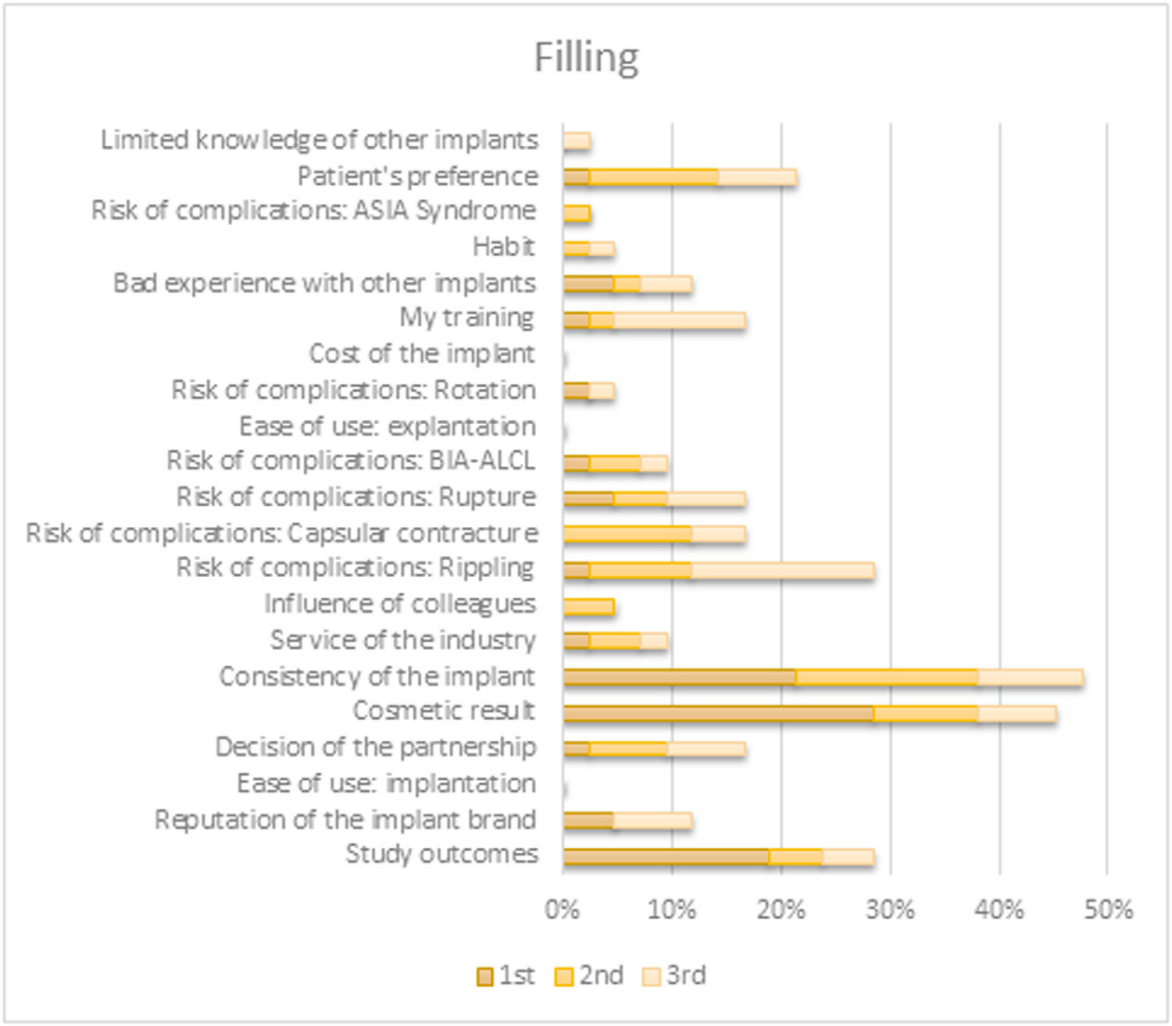
Survey respondents’ first, second, and third most important factor for determining their preferred filling. BIA-ALCL = Breast implant associated anaplastic large-cell lymphoma; ASIA = Autoimmune/inflammatory syndrome induced by adjuvants.

**Figure 6:**
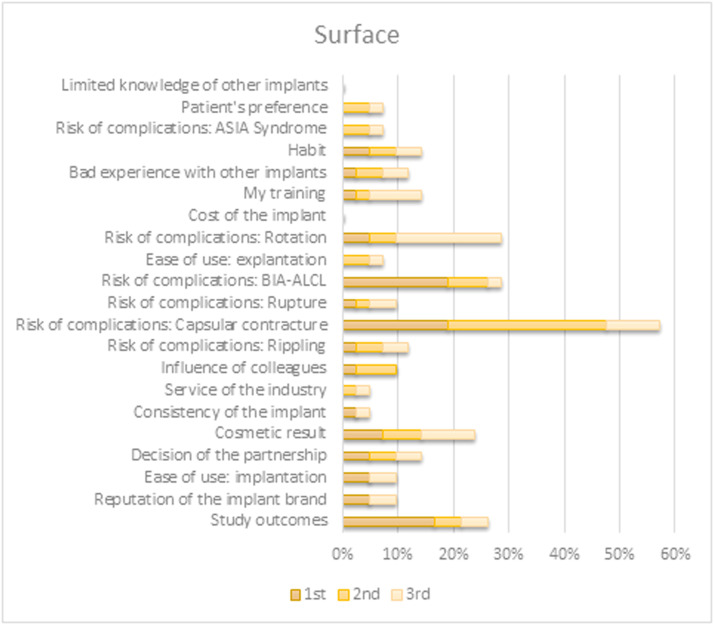
Survey respondents’ first, second, and third most important factor for determining their preferred surface. BIA-ALCL = Breast implant associated anaplastic large-cell lymphoma; ASIA = Autoimmune/inflammatory syndrome induced by adjuvants.

**Figure 7:**
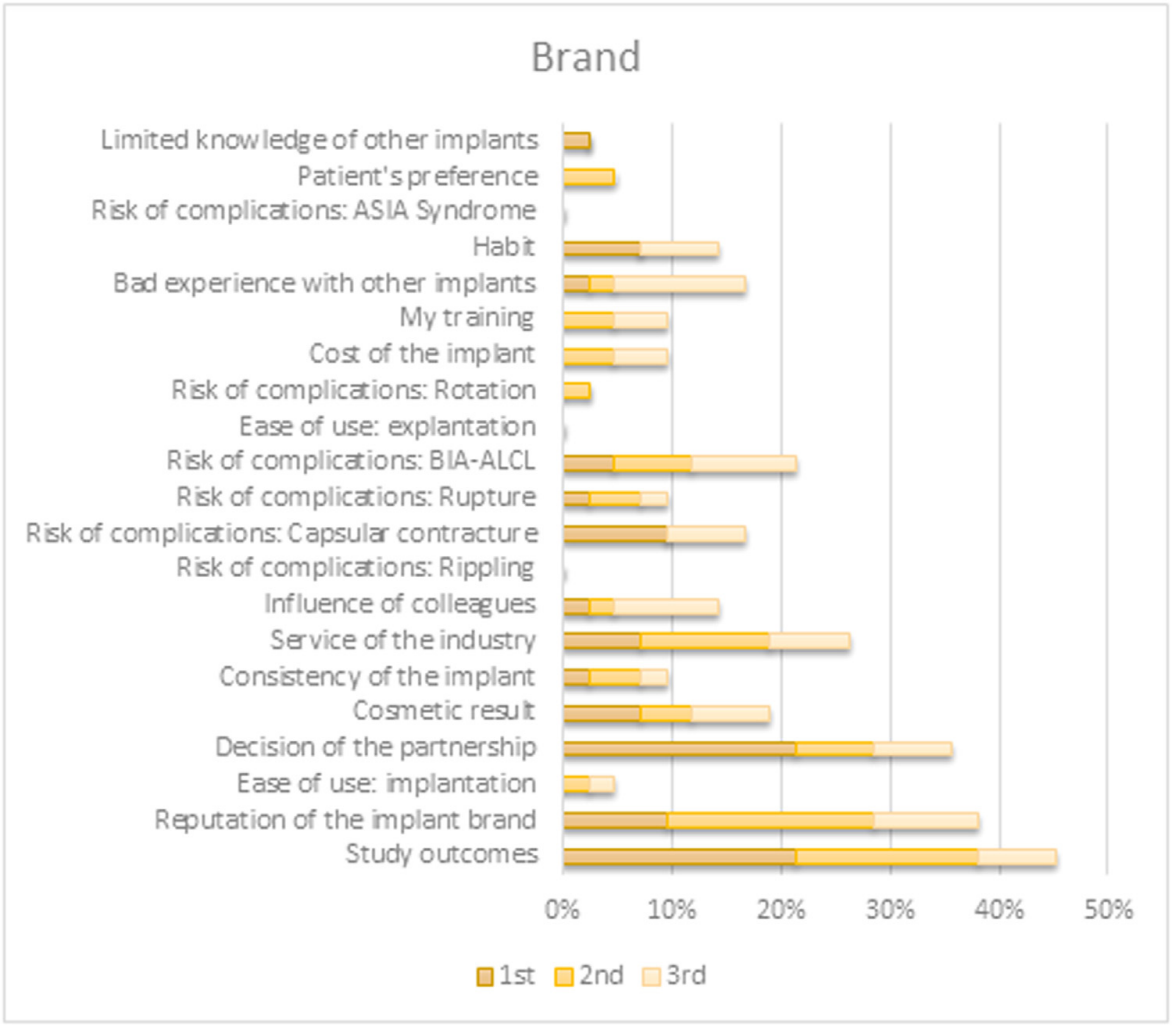
Survey respondents’ first, second, and third most important factor for determining their preferred brand. BIA-ALCL = Breast implant associated anaplastic large-cell lymphoma; ASIA = Autoimmune/inflammatory syndrome induced by adjuvants.

When prompted to select the top three factors directing their choice of a specific preferred implant characteristic, the outcomes differed between characteristics. Choices for both shape and filling were predominantly based on their contributions to the appearance and tactile qualities of the reconstruction. Preference for implant surface type originated primarily from (perceived) associated risks for various complications. The brand selection was based on study outcomes or brand reputation. An overview of all factors per implant characteristic is shown in Figure 4, Figure 5, Figure 6, Figure 7.

#### Indications for plastic surgeons to deviate from preferred breast implant

**Figure 8:**
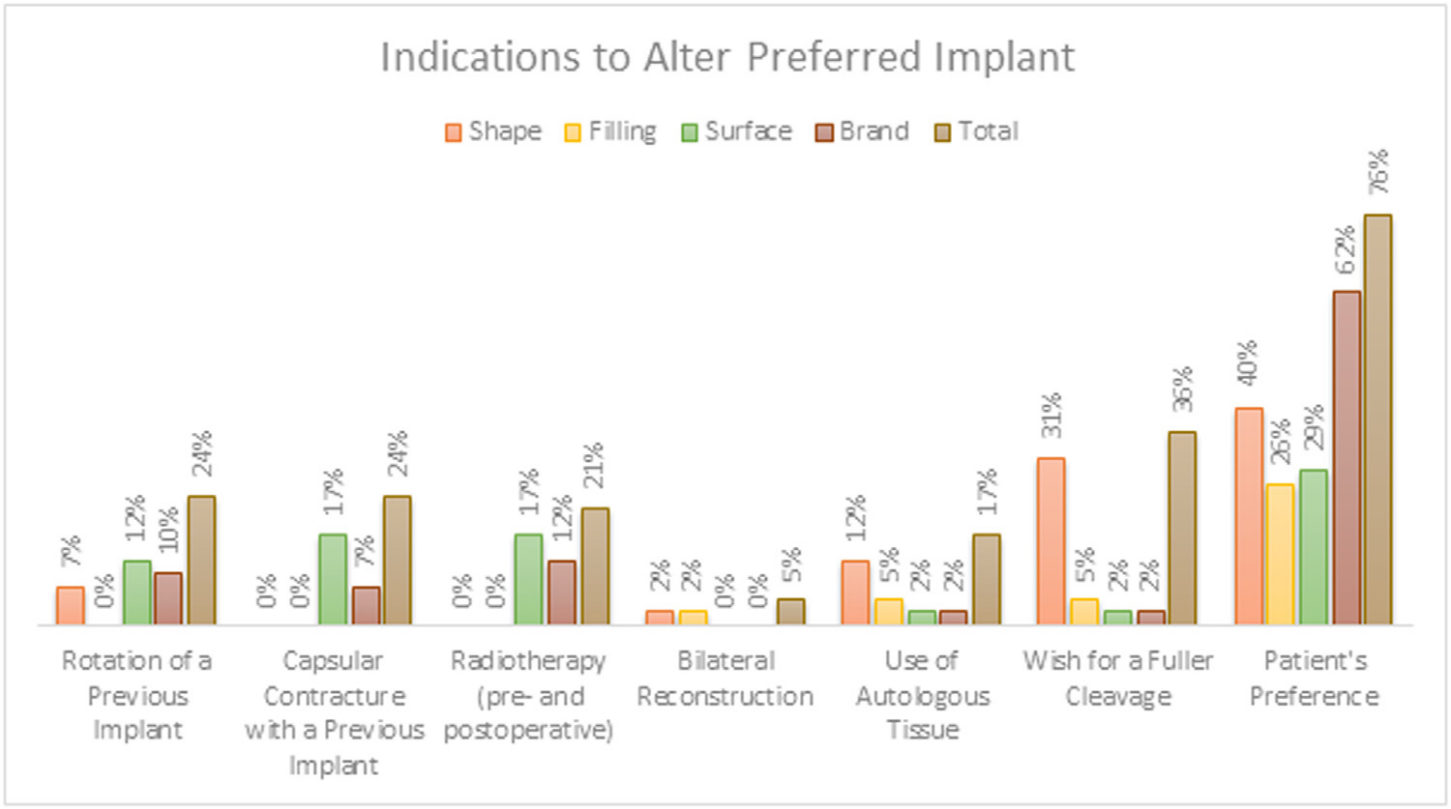
Percentage of survey respondents who would deviate from their preferred shape, filling, surface texture, and/or brand of breast implant given certain indications.

Ninety percent of the respondents were willing to deviate from their preferred implant for at least one of the indications presented (Figure 8). Eighteen respondents (43%) would not deviate from their preferred implant if a different type of breast implant was already in place in the contralateral breast.

Patients’ preference was found to be the main indication for plastic surgeons to deviate from their preferred breast implant.

## Discussion

This is the first study to provide an insight into all factors that plastic surgeons deem important when choosing an implant for breast reconstruction. Our two-step approach that used qualitative and quantitative components facilitated an extensive analysis of these factors. The interviews indicated various factors, including complication rates, marketing, economic and logistical factors, that influenced the choice of a breast implant. The results from the survey showed variation in preferred breast implant types, but also a substantial variation in factors were found to be important in choosing the implant. Furthermore, the valuation of factors differed when considering the choice of a preferred implant, overall, or per implant characteristic.

Overall, ‘‘study outcomes’’ was the most important factor, which is expected as this is a broad term that includes several other factors. Notably, the factor ‘‘reputation of the implant brand’’ was ranked as the second most important influencing factor. Reputation is not necessarily based on facts and is likely the result of marketing and media. It is known that media affects medical decision making^12^^,^^13^; however, the information delivered by media is not always true.^14^ Furthermore, previous work has shown that physicians tend be susceptible to marketing strategies, even if they believe their prescribing behavior is unaffected.^15^ The desire for high valuation of brand reputation is questionable, but it is reassuring that plastic surgeons are aware of this fact.

The media influence not only plastic surgeons but also patients. Especially in the current scenario, where social media popularity is high.^16^ This study showed that 76% of plastic surgeons were willing to deviate from their preferred implant to comply with the patient's preference. We did not specify the patient's preference when asking respondents if they would deviate from their preferred implant because of it. Nevertheless, we may assume that the media directly influence patients and consequently indirectly affect plastic surgeon's choice of breast implants.

In the Netherlands, several plastic surgeons working in general hospitals are involved in partnerships. Often, these partnerships jointly decide on the standard breast implant to be used and individual surgeons only have limited influence on this decision. Although the factor ‘‘decision of the partnership’’ was important to most respondents, a relatively large percentage strongly disagreed with this (10%), illustrating polarization. This factor represents a limited freedom of choice of breast implants because the availability was determined by the department, partnership, or hospital. When comparing respondents from academic and general hospitals, respondents from the latter ‘agreed’ or ‘strongly agreed’ more often (50% vs 65%, respectively). This might indicate that the freedom of choice in general hospitals is more restricted. A similar discrepancy was shown for the ‘‘cost of the implant’’. Only 13% of respondents working in academic hospitals ‘agreed’ or ‘strongly agreed’ this to be an important factor compared to 38% of respondents in general hospitals. A possible reason for this could be that, in the Netherlands, more than half of the specialists who work in general hospitals are self-employed.^17^ In this case, the cost of an implant directly affects their earnings. In academic hospitals, all specialists are in salaried employment and implant price does not affect them directly. Additionally, the factor ‘‘decision of partnership’’ was ranked fourth most important, suggesting that individual plastic surgeons had a limited say in choosing the available implants. This seems to contradict the vast majority of respondents stating they could influence the choice. Considering all this, plastic surgeons may not be as free to choose the best implant for their patients as they perceive. Also, patients may receive a different level of personalized medicine depending on the hospital at which they are treated.

When evaluating factors per implant characteristic, it was clear that certain aspects stand out in importance when choosing the shape, filling, or surface texture. The outliers for each were of a specific category, i.e., related to the appearance of the reconstruction for shape and filling and complication-related to the surface texture. When choosing implant surface, various complications proved to be important. The risk profile of each breast implant surface type is still under debate, and this is likely the case for other implant characteristics as well. This does not preclude these factors from weighing heavily when plastic surgeons determine their preferred implant. Notably, 8% of respondents, who preferred a textured implant, were not aware of type of texture (micro- or macro-). This appeared to be undesirable as recent literature distinguishes between degrees of roughness, which may correlate to different risk profiles.^18^^,^^19^ However, this survey was conducted before the influence of implant texturing in relation to the risk of BIA-ALCL received overwhelming media attention. We hypothesize that if we were to repeat the survey, the influence of surface texture would be ranked higher.

A previous study used a modified Delphi method to provide consensus recommendations on the key factors involved in implant selection for breast augmentation from the perspective of plastic surgeons who practiced in Australia and New Zealand.^8^ Although the authors focused on breast augmentation rather than reconstruction, their results provide insight into how plastic surgeons choose breast implants and how factors such as patient characteristics influence the selection. A panel of seven plastic surgeons was used to formulate specific recommendations regarding breast implant selection. Their aim was different from ours as we tried to identify all influencing factors and we investigated the considerations behind the various factors. Furthermore, we approached all plastic surgeons nationally, which resulted in a larger and potentially more representative sample size.

Given the uncertainties around implant risks mentioned by the media, patient requests may be difficult to refute based on scientific literature. Plastic surgeons are responsible for sharing their expertise with the patient owing to their experience and scientific knowledge. The importance of various complications in breast implant selection should ideally be determined via shared decision making between the patient and the surgeon, not by the surgeon alone. All significant complications and any uncertainty related to them and their potential consequences should be discussed clearly with the patient. Only then can we call it “informed consent.” We believe that the different types of breast implants should be discussed with the patient preoperatively, including implants that may not be the plastic surgeon's preference. Only then will patients be properly informed, resulting in true shared decision making. Notwithstanding, a surgeon's experience, training, and habit may rightly influence personal preferences, because familiarity with the implant used may contribute to a good and reproducible result.

Considering all the data obtained, it appears that plastic surgeons are not motivated by scientific evidence alone when choosing a breast implant. The extent to which marketing, media and social media affect their choice lies outside the scope of this study. Nevertheless, it raises doubts on whether this is ethically acceptable. The results of this study suggest that plastic surgeons are at least partly aware of the influence of these factors and it is important that our results reach plastic surgeons and increase awareness. To reduce the impact of these non-evidence based factors, plastic surgeons need high-quality information on breast implant safety, expected results, and complications. Therefore, we need high-quality research, and one of the best sources for this is an independent, prospective, nationwide database that tracks surgical outcomes of every breast implant used. The Netherlands is one of the first countries to have developed such an opt-out breast implant registry, namely the Dutch Breast Implant Registry.^1^ Every breast implant placed or removed in the Netherlands is registered in this database and all major surgical outcomes are recorded. Similar registries have been started in countries worldwide.^20^ This will support high-quality research that will lead to a better understanding of risk profiles associated with various breast implant characteristics.

A strength of this study was its mixed-method design. The qualitative interview study formed the basis for the creation of quantitative online survey study. A complete overview of all factors influencing the plastic surgeons’ decision making in implant selection was obtained from a widely varying population of plastic surgeons. Furthermore, the study population reasonably represented the target population.^11^

One limitation of this study was that only five plastic surgeons were interviewed to identify the factors influencing their choice of a breast implant in a reconstructive setting. Despite this small group, we expect that all factors were identified because data saturation occurred in the last two interviews. Another limitation was the low response rate of 13% to the online survey. Due to privacy laws in the Netherlands, it was not possible to send personal invitations or reminders to participants. However, all plastic surgeons in the Netherlands were invited to participate without preselecting reconstructive (breast) plastic surgeons. This could have led to an underestimation of the response rate. Furthermore, no validated questionnaire was used for the online survey. Additionally, right after the interviews and survey in 2018, silicone breast implants received increased media attention, which may have changed the valuation of the factors. Lastly, owing to the nature of this study, it was only possible to collect self-reported factors. However, part of a choice will also be made unconsciously. A discrete choice experiment could further investigate how breast implants are chosen.

## Conclusion

The list of self-reported factors influencing the plastic surgeons’ choice of a breast implant in a reconstructive setting is extensive. This study shows that scientific evidence is not the only factor influencing implant selection. Besides predictable factors like study outcomes and complication rates, marketing, economic, and logistic factors were also identified. The substantial variation in the weight attributed to complications indicates a lack of consensus on and interpretation of the risk profile of the various implant characteristics, which is facilitated by uncertainty in the scientific literature. Brand reputation is valuated highly by plastic surgeons, implying that media and marketing may have considerable influence on their decision making.

Given the public debate concerning breast implant safety and numerous available options, patients must be informed extensively during shared decision making to obtain true informed consent.

## Conflicts of interest

The Department of Plastic & Reconstructive Surgery of the Erasmus MC received funding from POLYTECH Health & Aesthetics GmbH as financial support for personnel costs for one PhD candidate (no grant number available) from July 2018 until July 2021. All remaining authors have declared no conflicts of interest.

## Appendix Supplementary materials

Image, application 1

Image, application 2

## References

[1] Rakhorst H.A., Mureau M.A.M., Cooter R.D. (2017). The new opt-out Dutch National Breast Implant Registry - Lessons learnt from the road to implementation. J Plast Reconstr Aesthet Surg.

[2] Nelson J.A., McCarthy C., Dabic S. (2021). BIA-ALCL and textured breast implants: A systematic review of evidence supporting surgical risk management strategies. Plast Reconstr Surg.

[3] Wohlgemuth F.B., Brasil M.B.Q., d'Acampora A.J (2019). Risk of breast implant-associated anaplastic large cell lymphoma in patients submitted to breast implantation: A systematic review. Breast J.

[4] Jara L.J., García-Collinot G., Medina G. (2017). Severe manifestations of autoimmune syndrome induced by adjuvants (Shoenfeld's syndrome). Immunol Res.

[5] Stiggelbout A.M., Pieterse A.H., De Haes J.C (2015). Shared decision making: Concepts, evidence, and practice. Patient Educ Couns.

[6] Shay L.A., Lafata J.E (2015). Where is the evidence? A systematic review of shared decision making and patient outcomes. Med Decis Making.

[7] Rocco N., Rispoli C., Moja L. (2016). Different types of implants for reconstructive breast surgery. Cochrane Database Syst Rev.

[8] Magnusson M.R., Connell T., Miroshnik M. (2019). Breast implant selection: Consensus recommendations using a modified Delphi method. Plast Reconstr Surg Glob Open.

[9] Adams W.P., McKee D (2016). Matching the implant to the breast: A systematic review of implant size selection systems for breast augmentation. Plast Reconstr Surg.

[10] Tong A., Sainsbury P., Craig J (2007). Consolidated criteria for reporting qualitative research (COREQ): A 32-item checklist for interviews and focus groups. Int J Qual Health Care.

[11] Netherlands Society for Plastic Surgery (NVPC). Members survey. 2017.

[12] Corbett J.B., Mori M (1999). Medicine, media, and celebrities: News coverage of breast cancer, 1960-1995. Journalism Mass Commun Q.

[13] Williams D., Kelly A., Feely J (2000). Influence of media and regulatory changes on prescribing of cotrimoxazole and trimethoprim in Ireland. Pharmacoepidemiol Drug Saf.

[14] Cipriani C., Pepe J., Minisola S., Lewiecki E.M (2018). Adverse effects of media reports on the treatment of osteoporosis. J Endocrinol Invest.

[15] Sah S., Fugh-Berman A (2013). Physicians under the influence: Social psychology and industry marketing strategies. J Law Med Ethics.

[16] Moorhead S.A., Hazlett D.E., Harrison L., Carroll J.K., Irwin A., Hoving C (2013). A new dimension of health care: Systematic review of the uses, benefits, and limitations of social media for health communication. J Med Internet Res.

[17] (2021). Federatie medisch specialisten. Feiten Cijfers Med Specialistische Zorg.

[18] Mempin M., Hu H., Chowdhury D., Deva A., Vickery K. (2018). The A. B and C's of silicone breast implants: Anaplastic large cell lymphoma, biofilm and capsular contracture. Materials.

[19] Munhoz A.M., Clemens M.W., Nahabedian M.Y (2019). Breast implant surfaces and their impact on current practices: Where we are now and where are we going?. Plast Reconstr Surg Glob Open.

[20] Spronk P.E.R., Begum H., Vishwanath S. (2020). Toward international harmonization of breast implant registries: International collaboration of breast registry activities global common data set. Plast Reconstr Surg.

