# Supplementary material for: Decision making in breast implant selection for breast reconstruction: A mixed-method study among plastic surgeons
Source: JPRAS Open. 2023 Oct 21;38:283–95. doi: 10.1016/j.jpra.2023.10.009 (PMC10663762; doi:10.1016/j.jpra.2023.10.009)
Supplement: Supplementary file 1 [file mmc1.docx]

# Appendix A: Predefined structure for the interviews

1. Since when have you been a plastic surgeon?
2. What types of implants did you work with during your training?
3. Since when have you been doing breast reconstructions independently?
4. Approximately how many breast reconstructions with implants do you do per year?
5. How many breast reconstructions with implants are done in your hospital per year?
6. Who decides which implants are available to you?
7. What type of implant do you use the most?
8. Are there types of implants that are not available to you?
9. Which factors influence your choice for an implant? Think as broadly as possible, including non-clinical factors.
   1. How do these factors influence that choice?
   2. Which are the most influential?
10. In your opinion, are other reconstructive surgeons influenced by other factors?
11. What different types of brand do you use and in what proportion?
    1. So you use [X] the most. Why is that?
    2. Are there any drawbacks to this type?
    3. When do you choose another brand?
    4. So you use [Y] the least/rarely. Why is that?
    5. Are there any advantages to this type?
12. What different types of shape do you use and in what proportion?
    1. So you use [X] the most. Why is that?
    2. Are there any drawbacks to this type?
    3. When do you choose another shape?
    4. So you use [Y] the least/rarely. Why is that?
    5. Are there any advantages to this type?
13. What different types of filling do you use and in what proportion?
    1. So you use [X] the most. Why is that?
    2. Are there any drawbacks to this type?
    3. When do you choose another filling?
    4. So you use [Y] the least/rarely. Why is that?
    5. Are there any advantages to this type?
14. What different types of surface do you use and in what proportion?
    1. So you use [X] the most. Why is that?
    2. Are there any drawbacks to this type?
    3. When do you choose another surface?
    4. So you use [Y] the least/rarely. Why is that?
    5. Are there any advantages to this type?
15. I can imagine that these 4 characteristics also influence each other. For example, with an anatomical implant you are more likely to opt for a textured surface. Are there such connections for you?
