# Supplementary material for: Decision making in breast implant selection for breast reconstruction: A mixed-method study among plastic surgeons
Source: JPRAS Open. 2023 Oct 21;38:283–95. doi: 10.1016/j.jpra.2023.10.009 (PMC10663762; doi:10.1016/j.jpra.2023.10.009)
Supplement: Supplementary file 2 [file mmc2.docx]

# Appendix B: Digital survey

1. What is your gender?
   1. Male
   2. Female
2. What is your age?
   1. …
3. Where did you do your training?
   1. Amsterdam (VU)
   2. Amsterdam (AMC)
   3. Rotterdam
   4. Groningen
   5. Nijmegen
   6. Zwolle
   7. Leeuwarden
   8. Maastricht
   9. Eindhoven
   10. Utrecht
   11. Other: …
4. How many years have you been a plastic surgeon?
   1. …
5. At what hospital type do you work mainly?
   1. Community hospital
   2. Academic hospital
6. What type of reconstruction do you perform most?
   1. Direct-To-Implant
   2. Two-stage reconstruction using a tissue-expander
7. Do you also perform breast augmentation without medical indication?
   1. Yes, less than reconstructions
   2. Yes, as much as reconstructions
   3. Yes, more than reconstructions
   4. No
8. How many unique reconstructions do you perform per year?
   1. …
9. Did you have influence on current default breast implant?
   1. Yes
   2. No
10. Which shape do you currently use most?
    1. Anatomical
    2. Round
11. Which filling do you currently use most?
    1. Saline
    2. Silicone
    3. Hydrogel
12. Which surface do you currently use most?
    1. Smooth
    2. Textured
       - Microtexturing
       - Macrotexturing
       - I don’t know
    3. Poly-urethane coating
13. Which brand do you currently use most?
    1. Mentor
    2. Allergan
    3. Motiva
    4. Eurosilicone
    5. Polytech
    6. Silimed
    7. Negor
    8. B-Lite
    9. Monobloc
    10. Other: …
14. Statement: The factors below have led to the choice of my preferred implant.

Please indicate below to what extent you agree or disagree with this statement.

|  | Totally unagree | Unagree | Somewhat unagree | Somewhat agree | Agree | Totally agree |
| --- | --- | --- | --- | --- | --- | --- |
| Ease of use: implantation | O | O | O | O | O | O |
| Ease of use: explantation | O | O | O | O | O | O |
| Cost of the implant | O | O | O | O | O | O |
| Study outcomes | O | O | O | O | O | O |
| Patient’s preference | O | O | O | O | O | O |
| Cosmetic result | O | O | O | O | O | O |
| Consistency of the implant (form-retaining/feel) | O | O | O | O | O | O |
| Service of the industry | O | O | O | O | O | O |
| Reputation of the implant brand | O | O | O | O | O | O |
| Risk of complications: BIA-ALCL | O | O | O | O | O | O |
| Risk of complications: rupture | O | O | O | O | O | O |
| Risk of complications: ASIA/BII | O | O | O | O | O | O |
| Risk of complications: rotation / displacement | O | O | O | O | O | O |
| Risk of complications: capsular contracture | O | O | O | O | O | O |
| Risk of complications: rippling | O | O | O | O | O | O |
| Decision of the partnership | O | O | O | O | O | O |
| Opinions of colleagues in the field | O | O | O | O | O | O |
| Plastic surgical training | O | O | O | O | O | O |
| Habit | O | O | O | O | O | O |
| Limited knowledge of other implants | O | O | O | O | O | O |
| Bad experience with other implants | O | O | O | O | O | O |

Below is the list of factors, but now numbered. The next four questions will prompt you to choose from this list.

1. Ease of use: implantation

2. Ease of use: explantation

3. Cost of the implant

4. Study outcomes

5. Patient’s preference

6. Cosmetic result

7. Consistency of the implant (form-retaining/feel)

8. Service of the industry

9. Reputation of the implant brand

10. Risk of complications: BIA-ALCL

11. Risk of complications: rupture

12. Risk of complications: ASIA/BII

13. Risk of complications: rotation / displacement

14. Risk of complications: capsular contracture

15. Risk of complications: rippling

16. Decision of the partnership

17. Opinions of colleagues in the field

18. Plastic surgical training

19. Habit

20. Limited knowledge of other implants

21. Bad experience with other implants

1. Which three factors are most important when choosing the **shape** of your preferred implant? Choose those three factors from the list above and put them in order of importance, with 1 being the most important.
   1. First most important factor: …
   2. Second most important factor: …
   3. Third most important factor: …
2. Which three factors are most important when choosing the **filling** of your preferred implant? Choose those three factors from the list above and put them in order of importance, with 1 being the most important.
   1. First most important factor: …
   2. Second most important factor: …
   3. Third most important factor: …
3. Which three factors are most important when choosing the **surface** of your preferred implant? Choose those three factors from the list above and put them in order of importance, with 1 being the most important.
   1. First most important factor: …
   2. Second most important factor: …
   3. Third most important factor: …
4. Which three factors are most important when choosing the **brand** of your preferred implant? Choose those three factors from the list above and put them in order of importance, with 1 being the most important.
   1. First most important factor: …
   2. Second most important factor: …
   3. Third most important factor: …
5. Do you deviate from your preferred implant when following indications occur? If yes, which characteristic do you deviate from?

Rotation / malposition of previous implant

- Yes
  - Shape
  - Filling
  - Surface
  - Brand
- No

Capsular contracture of previous implant

- Yes
  - Shape
  - Filling
  - Surface
  - Brand
- No

Preoperative or postoperative radiotherapy

- Yes
  - Shape
  - Filling
  - Surface
  - Brand
- No

Bilateral breast reconstruction

- Yes
  - Shape
  - Filling
  - Surface
  - Brand
- No

Using autologous tissue (for example, an LD prosthesis reconstruction)

- Yes
  - Shape
  - Filling
  - Surface
  - Brand
- No

Wish for a fuller cleavage

- Yes
  - Shape
  - Filling
  - Surface
  - Brand
- No

Presence of another implant in the contralateral breast

- Yes
  - Shape
  - Filling
  - Surface
  - Brand
- No

Patient’s preference

- Yes
  - Shape
  - Filling
  - Surface
  - Brand
- No
